# Understanding the patient-experienced trajectory of sleep across the hematopoietic cell transplantation process

**DOI:** 10.1007/s00520-026-11365-8

**Published:** 2026-11-06

**Authors:** Gabriella Sanabria, Lianel P. Rosario, KD L. Jacobs, Melody N. Chavez, Brandy L. Arredondo, Carley Geiss, Taylor Welniak, Yvelise Rodriguez, Sheila N. Garland, Eric S. Zhou, Heather S. L. Jim, Laura B. Oswald, Asmita Mishra, Joseph Pidala, Brian D. Gonzalez

**Affiliations:** 1 Department of Health Outcomes and Behavior, Moffitt Cancer Center, 12902 Magnolia Drive, MRC-HOB, Tampa, FL 33612 USA; 2 Ponce Health Sciences University, Ponce, PR USA; 3 College of Public Health, University of South Florida, Tampa, FL USA; 4 Present Address: ICF, Reston, VA USA; 5 Participant Research, Interventions, and Measurement Core Facility, Moffitt Cancer Center, Tampa, FL USA; 6 Department of Psychology, Memorial University, St. John’s, NL Canada; 7 Department of Oncology, Memorial University, St. John’s, NL Canada; 8 Department of Supportive Oncology, Dana-Farber Cancer Institute, Boston, MA USA; 9 Blood and Marrow Transplant and Cellular Immunotherapy, Moffitt Cancer Center, Tampa, FL USA; 10 Department of Oncologic Science, University of South Florida, Tampa, FL USA

**Keywords:** Hematopoietic cell transplantation, Sleep disturbance, Quality of life, Survivorship

## Abstract

**Purpose:**

Sleep disturbance is strongly associated with reduced quality of life and increased anxiety and depression. However, research on what contributes to these negative biobehavioral symptoms among hematopoietic cell transplantation (HCT) recipients remains limited. This study sought to understand sleep disturbances experienced by allogeneic HCT survivors from the perspectives of patients, clinicians, and caregivers, to identify key contributors to sleep disturbances across the HCT trajectory.

**Methods:**

This cross-sectional qualitative study included semi-structured interviews with a purposive sample of HCT survivors, caregivers, and clinicians. Interview data were analyzed using an applied thematic approach.

**Results:**

Participants (*N* = 29) were mostly female (69%), White (90%), and non-Hispanic (86%). Clinically relevant time periods emerged in patients’ journeys that mark changes in their sleep patterns: during hospitalization, the isolation period after hospitalization, and at home after isolation (i.e., around day +90). In each period, cross-cutting themes emerged that present the trajectory of patients’ sleep disturbance throughout the HCT process. Patients continued with poor maladaptive sleep habits acquired during hospitalization after returning home.

**Conclusion:**

This study expands research on biobehavioral symptoms among HCT recipients, providing context for quantitative findings in the literature and helping to map the sleep trajectory of allogeneic HCT recipients from multiple perspectives. Future research should consider using the patient, clinician, and caregiver perspectives presented in this work to identify points for targeted interventions that improve patients’ sleep and overall quality of life and to advocate for “sleep-friendly” hospital procedures before chronic sleep issues become entrenched.

## Introduction

Hematopoietic cell transplantation (HCT) is a widely used treatment that can offer the best chance of survival for patients with hematologic malignancies and other disorders [1]. More than 23,000 HCTs were performed in the USA in 2023, with the HCT survivorship population projected to reach 500,000 by 2030 [1–4]. As this population grows, understanding the potential negative, lingering biobehavioral symptoms (i.e., patient-reported physical, cognitive, and psychological symptoms) experienced by HCT patients is crucial to inform efforts to improve their quality of life (QOL) and adjustment post-transplant.

Sleep disturbance is a common lingering biobehavioral symptom reflecting a dysregulation of homeostatic sleep drive, circadian rhythms, and stress-related neuroendocrine and inflammatory pathways [5]. It encompasses difficulties in initiating and maintaining sleep, including interruptions to the sleep-wake schedule, and waking up fatigued [6]. Among HCT survivors, sleep disturbance can shift from relatively mild before transplant (e.g., occasional daytime drowsiness) to profound (e.g., dysregulated sleep-wake cycles) post-transplant [7–9]. An estimated 77% of patients report experiencing sleep disturbance during hospitalization, in part due to hospital noise and rounding schedule [10]. Although common pre-transplant, sleep disturbance frequently worsens in severity post-transplant and persists in up to 43% of HCT survivors, due to overlapping acute side effects and lingering negative sleep patterns from the first 100 days post-transplant [7–9, 11, 12]. Sleep disturbance becomes chronic when sleep difficulties occur at least three nights per week and last for more than 3 months [13]. Studies indicate that sleep disturbance is strongly associated with several negative outcomes, including worse QOL, anxiety, depression, lower adherence to oral medications, and worse overall survival [11, 12, 14]. However, sleep disturbance is often overlooked by clinicians, with sleep patterns and sleep disruption rarely included in patient communication [15].

The majority of studies assessing sleep in HCT survivors focus on quantitative assessments (e.g., Insomnia Severity Index, Pittsburgh Sleep Quality Index) and do not contextualize findings within patients’ experiences, which could help explain factors contributing to these negative biobehavioral symptoms [7, 12]. This qualitative study aimed to characterize sleep disturbances experienced by allogeneic HCT recipients, bringing the perspectives of patients, clinicians, and caregivers to identify key contributors to sleep disturbance across the HCT trajectory. This study focuses on allogeneic HCT recipients, as they may be particularly vulnerable to frequent and severe sleep disturbance due to longer recovery trajectories and greater symptom burden [7].

## Methods

### Study sample

Using purposive sampling, caregivers, HCT recipients, and clinicians were recruited for the study. Eligible HCT recipients were adults aged ≥ 18 years who had undergone allogeneic HCT for a hematologic malignancy within the previous 6–24 months and self-reported clinically significant sleep disturbance (a score of ≥ 4 on a 0–10 severity scale). Individuals with documented severe and untreated neurological or psychiatric disorders that could interfere with participation were excluded. Additional inclusion criteria included the ability to speak and read English and provide informed consent. Caregivers were eligible if they were ≥ 18 years, were able to speak and read English, and self-identified as a primary caregiver for an HCT recipient. Clinicians’ only eligibility criterion was being members of Moffitt Cancer Center’s (MCC) Blood and Marrow Transplant Program.

Potentially eligible HCT recipients were identified from electronic medical records at MCC and approached in person during a routine outpatient appointment at MCC or via telephone. Eligible patients were asked to nominate and provide contact information for their primary caregiver before, during, or after their HCT hospitalization. Caregivers nominated by enrolled patients were approached either in person (e.g., the outpatient appointment) or via telephone. Clinicians affiliated with MCC’s Blood and Marrow Transplant Program were approached in person, via telephone, or e-mail. Participating clinicians included oncologists (*n* = 4), nurse coordinators (*n* = 2), a nurse practitioner (*n* = 1), an interventional radiology technician (*n* = 1), and a physician assistant (*n* = 1).

Advarra Institutional Review Board approved all study activities (MCC 19486, MCC 23826, Pro00024945). All eligible participants completed informed consent before participating in any research activity and received a $50 gift card as compensation for their time.

### Data collection

Members of the study team conducted the semi-structured interviews from May to August 2018. The study had 29 participants: *n *= 10 HCT recipients, *n* = 10 caregivers of HCT recipients, and *n* = 9 clinicians. The sample size was informed by thematic saturation principles and existing qualitative research, indicating saturation is typically reached within 9–17 interviews among relatively homogeneous samples [16, 17]. The same semi-structured interview guide was used for each subgroup; however, the questions were tailored to the group being interviewed (Table 1). The interview guide explored the following domains: (1) the trajectory of HCT recipients’ sleep disturbance from before the HCT, during HCT hospitalization, and in the survivorship phase; (2) strategies to manage barriers to sleep.

**Table 1:** Interview domains and example questions

Interview domain	Example interview question
The trajectory of HCT recipients’ sleep disturbance from before the HCT, during HCT hospitalization, and in the survivorship phase	• Could you describe what your sleep was like before the transplant? • Could you describe what your sleep was like during the hospitalization? • Could you describe what your sleep was like shortly after you went home from the hospital? o *Probe*: And in the time since? • Did you sleep during the day while you were in the hospital for the transplant? ⚬ *Probe*: Why/why not do you think that was? ⚬ *Probe*: Can you tell me more about that? • Did you sleep during the day shortly after you went home from the hospital? ⚬ *Probe*: Can you tell me more about that? ⚬ *Probe*: What about in the months after that time?
Strategies to manage barriers to sleep	• What do you think caused your sleep to worsen? • Did you take any steps to improve your sleep? ⚬ *Probe*: What were they? ⚬ *Probe*: Did they help?

Note. We replaced “you” with “your loved one” or “your patients” as needed for caregivers and clinicians

BG and a trained staff member conducted interviews in person or remotely via a secure call, based on participant preference. Interviews lasted on average 35 min. All interviews were audio-recorded and uploaded to NVivo Transcription for automated transcription [18]. Trained staff (MC and BA) manually reviewed and corrected all transcripts to ensure accuracy. Finalized verbatim transcripts were uploaded to Dedoose (version 9.0.17), a cloud-based analysis platform [19]. All demographic information was obtained after informed consent, but before the interviews were conducted via chart review for patients and caregivers and self-report for clinicians. Clinical information was abstracted from patients’ electronic medical records.

### Data analysis

Two trained qualitative researchers (MC and BA) developed a preliminary codebook using Microsoft Excel. Initial codes were derived a priori from the interview guide domains, with additional codes incorporated based on emergent themes identified during transcript review. The codebook was refined through multiple rounds of iterative coding and agreement testing to achieve acceptable intercoder reliability (Cohen’s kappa = 0.82) [20]. Discrepancies were resolved by consensus. Transcripts were then coded using Dedoose analysis software. Applied thematic analysis techniques were used to identify patterns related to sleep disturbance and its contributing factors [21].

To maintain the qualitative rigor of our study, we engaged in various validated techniques, including addressing reflexivity and performing an iterative coding process with ongoing communication between coders to refine definitions and add new codes based on emerging data [22, 23]. Confirmability and dependability were enriched through the audit trail describing the coding and analysis process and the multiple rounds of intercoder agreement, updating the codebook to match the reached consensus [23, 24]. Finally, the thorough description of the inclusion criteria helps to address the transferability of the study [24].

## Results

### Study sample characteristics

Participants (*N* = 29) consisted of 10 HCT recipients (34.5%), 10 HCT caregivers (34.5%), and 9 HCT clinicians (31.0%, Table 2). HCT recipients were being treated for acute myeloid leukemia (AML; *n* = 5), acute lymphoblastic leukemia (ALL; *n *= 2), myelodysplastic syndrome (MDS; *n* = 1), aplastic anemia (AA; *n* = 1), and chronic myelomonocytic leukemia (CMML; *n* = 1). The mean age for HCT recipients was 54.2 years (SD = 4.5, range = 48–61) and the mean amount of time that had passed from HCT to the interview was 16.2 months (SD = 5.4, range = 6.2–21.6).

**Table 2:** Participant characteristics (*N *= 29)

	Patients (*N *= 10)		Caregivers (*N* = 10)		Clinicians (*N* = 9)	
	*N*	%	*N*	%	*N*	%
Sex						
Female	6	60	7	70	7	78
Male	4	40	3	30	2	22
Ethnicity						
Hispanic	2	20	1	10	1	11
Non-Hispanic	8	80	9	90	8	89
Race						
White	9	90	10	100	7	78
Asian	0	0	0	0	2	22
Unknown	1	10	0	0	0	0

### Trajectory of sleep disturbance

Three clinically relevant time periods in the patients’ journeys marking changes in their sleep patterns—during hospitalization for HCT, the isolation period after hospitalization (typically through day 90), and home after isolation (after day 90)—were discussed in the interviews. In each period, cross-cutting themes surfaced that characterize the trajectory of patients’ sleep disturbance throughout the HCT process from the patient, caregiver, and clinician perspectives. Table 3 summarizes the HCT transplant trajectory and major themes at each stage.

**Table 3:** Summary: major themes throughout the transplant trajectory

Stage in transplant trajectory	Major themes
During hospitalization	• Challenges adjusting to the hospital schedule • Environmental factors negatively contributing to poor sleep habits • Treatment side effects negatively impacting quality of sleep • Anxiety and loss of personal control resulting in sleep disturbances
Isolation period after hospitalization (through day 90)	• Continuation of poor sleep habits acquired during hospitalization • Lingering treatment side effects • Challenges being in an unfamiliar, temporary place • Worry about adjusting to life post-transplant and the “new normal”
Home after isolation (post day 90)	• Improved, but lingering, sleep disturbance in the home environment • Lingering treatment toxicities • Continued worry about adjusting to life post-transplant and the “new normal”

### During hospitalization

Patients, caregivers, and clinicians overwhelmingly agreed that the hospitalization time period is when sleep was most disrupted. Together, they attributed patients’ inadequate sleep to four major themes: (1) challenges adjusting to hospital schedule; (2) environmental factors negatively contributing to poor sleep habits; (3) treatment side effects negatively impacting quality of sleep; (4) anxiety and loss of personal control resulting in sleep disturbances. Table 4 presents representative quotes illustrating the themes discussed below.

**Table 4:** Main themes and representative quotes: during hospitalization

Main themes	Representative quotes (participant, sex)
Theme 1: Challenges adjusting to the hospital schedule	“…once you were to fall asleep, something would come up, and you’d get woken up again.” (MDS patient #100, female)
	“She was tired all the time. And so, if she’s sleeping all day and not getting much exercise, then she wasn’t sleeping well at night. We were trying to walk around… the hospital in the transplant wing. Every day we walked several laps up to maybe 12, 14 laps a day. But then there were days that she couldn’t do that at all. All she wanted to do was sleep.” (MDS caregiver #208, female)
	“Sometimes they’re so turned around from being in the hospital. And when they’re in the hospital, they’re awakened every couple of hours for nurses and vitals and this and that and the clinicians. And then, so their sleep cycle gets completely flip flopped. And so sometimes they struggle with trying to reverse that, and they must try to keep themselves up during the day so they can sleep at night.” (physician assistant #300, female)
Theme 2: Environmental factors negatively contributing to poor sleep habits	“Once he had the transplant, just being in the bed all that time, you know, those beds are tough to take.” (CMML caregiver #203, female)
	“I mean, other than trying to make the hospital more comfortable. I know one of my things that always annoyed me was when I was trying to sleep, and the nurses were right outside at the nurse’s station talking, and that one that made it hard. But at the same time, you know that they’re doing their job and, you know, you can’t just get up and go, ‘Hush.’” (AML patient #102, female)
	“But the lights, the noise, the staff, being in a foreign area, the beds all contribute to just poor sleep habits. So that has even led us to give them a sleep aid pretty much reflexively while they’re in the hospital.” (interventional radiology technician #303, female)
Theme 3: Treatment side effects negatively impacting quality of sleep	“So, they don’t get enough sleep or their sleep gets interrupted because they’re having certain symptoms, such as the nausea, vomiting…at that night or they have diarrhea.” (oncologist #305, female)
	“Even the medications, obviously, we must be on them. But even they interrupt your sleep and their quality.” (AML patient #102, female)
	“For the transplant, he wasn’t sleeping because I guess he was in pain, nauseated, feeling sick, and you know all that stuff. He would sleep. He would just keep waking up because all the symptoms that he kept having.” (ALL caregiver #201, female)
Theme 4: Anxiety and loss of personal control resulting in sleep disturbances	“I still had racing thoughts throughout the hospital process. But, you know, because there is still so much unknown, what are we doing or is it going what is happening? You know, ‘How’s my body going to react to this medication, that chemotherapy?’ You know, ‘What’s going to happen with my job?’ You know, all those things went through my head while I was in the hospital still.” (AML patient #102, female)
	“When you’re stuck in a hospital, you lose control of what’s going on around you, right? She would not do that. She would not release that control…And she just, she was the financial adviser for the college though. So, you know, she was in charge. She’s you know, she’s, wanted to be in charge herself and stuff like that. This kind of hit her hard. Hit me hard, too.” (AML caregiver #200, male)

#### Theme 1: Challenges adjusting to hospital schedule

With vitals taken every four hours and labs and scans sometimes scheduled in the middle of the night, patients relied on “catnaps” to catch up on sleep, with most patients describing their sleep as “horrible” or “very interrupted.” The disruptive vital sign checks and treatment schedules caused adjustment issues, as patients were forced to alter their sleep cycles rapidly and radically. The patients could not keep themselves awake throughout the day to maintain a sleep cycle similar to their pre-hospitalization pattern. The clinicians also shared similar stories about the difficulties adjusting to the hospital schedule.

#### Theme 2: Environmental factors negatively contributing to poor sleep habits

Beyond adjusting to the hospital schedule, environmental factors such as lights, beds, noise, temperature, pillows, and being in an unfamiliar space also interfered with patients’ sleep. Caregivers and patients both commented on how the various combinations of these factors prevented patients from being comfortable and, for some, added to their anxiety. Patients shared how sleep was difficult with the typical sounds of a hospital*.* Clinicians also noted the negative impact of this hospital environment throughout their interviews. They most often cited the hospital setting as a contributor to patients’ poor sleep schedules, leading them to prescribe sleeping aids.

#### Theme 3: Treatment side effects negatively impacting quality of sleep

In addition to the impact of the new hospital environments, chemotherapy and medications caused gastrointestinal (GI) side effects, such as nausea and diarrhea, that frequently awoke patients throughout the night to use the bathroom, further compounding sleep disturbance. Additionally, the steroids commonly prescribed to transplant patients caused difficulty falling and staying asleep. Clinicians, patients, and caregivers echoed the negative impact of treatment side effects on the patients’ sleep quality.

#### Theme 4: Anxiety and loss of personal control resulting in sleep disturbances

During the hospitalization, the immense amount of clinical activity combined with the stress of being sick overwhelmed patients, making it difficult for them to feel as if they fully comprehended the transplant process and had any personal control during the process. Patients experienced anxiety about potential treatments, their future health, and the impact their illness will have on their jobs. This anxiety led to difficulty falling asleep or returning to sleep after overnight awakenings. Even when they could sleep, caregivers witnessed the patients’ restless sleep due to anxiety and their weakening grasp of personal control.

### Isolation period after hospitalization (through day 90)

Post-transplant, HCT recipients must remain in protective isolation, maintaining strict infection-prevention measures, with only the patient and caregiver present during the early post-transplant period (through day 90) due to their severely weakened immune system and high risk of infection. In our study, eight out of ten patients stayed in a temporary lodging during the isolation period. During the isolation period, the majority of patients agreed they continued to have sleep difficulties even after leaving the hospital. Many of the reasons for their sleep disturbances were similar to and extensions of those seen during hospitalization. Four major recurring themes surrounding inadequate sleep during this time period were the following: (1) continuation of poor sleep habits acquired during hospitalization; (2) lingering treatment side effects; (3) challenges being in an unfamiliar, temporary place; (4) worry about adjusting to life post-transplant and the “new normal.” Representative quotes illustrating the themes depicted below are presented in Table 5.

**Table 5:** Main themes and representative quotes: isolation period after hospitalization

Main themes	Representative quotes (participant, sex)
Theme 1: Continuation of poor sleep habits acquired during hospitalization	“I think a lot of times soon after, I think that their sleep-wake has been so disturbing from being inpatient that a lot of them have hard time developing routine patterns when they get out. They still, a lot of them, still nap during the day because their activity level inpatient was so poor and they come out deconditioned, even young people. That they end up, I think, almost maybe resting too much during the day and probably going to bed too early. So very, you know, it’s not uncommon to hear people say, ‘Oh, I wake up at 2:00, 3:00 in the morning and I can’t get back to sleep,’ or ‘I get back to sleep at 7 o’clock and then I sleep till 11:00.’” (nurse practitioner #301, female)
Theme 2: Lingering treatment side effects	“Initially…for about a month. I had a rash, but I didn’t, they didn’t keep me from sleeping. But then when the GI symptoms started, that was when I had a lot of difficulty sleeping…I could go to when I could fall asleep easily. But it didn’t. It didn’t last for long. I have to get up because of the symptoms…I just remember that time being so fatigued by the multiple trips to the bathroom. That’s why I think I was able to fall back asleep quite easily.” (AML patient #103, female)
	“Well, after the transplant, he wasn’t doing good either because he was so sick. He was so sick; I always have to help. I will give him his medication all the time, and he was always nauseated. He was always feeling sick and will always have to help him to get up from the bed and go to the bathroom.” (ALL caregiver #201, female)
	“Several of the patients are…continue to be on steroids now for graft versus host disease-related issues. And that continues to be an area of concern because their sleep does get disturbed on steroids, particularly when they’re at the higher doses. They’re completely agitated from that.” (oncologist #306, female)
Theme 3: Challenges being in an unfamiliar, temporary place	“So, I was staying in a hotel and of course, staying in a hotel. You’re not comfortable. So that that was probably a little better than being in the hospital, but not that much different because you’re still not in a comfortable environment again. Necessary…But of course, it’s still uncomfortable…” (AML patient #102, female)
	“It was harder to sleep. You know, we were in a strange apartment. It was an apartment complex. There was noise, you know. Your…habits are…even your TV viewing isn’t the same because it’s different stations and you don’t have the same things available too, you know.” (CMML patient #105, male)
Theme 4: Worry about adjusting to life post-transplant and the “new normal”	“But after I said after two months. I stopped sleeping pretty much. I mean, I wouldn’t take medications, I wouldn’t take anything to sleep even though I was given Valium because I was diagnosed with PTSD. I don’t, I don’t take them. I really, unless I have a bad anxiety attack, I don’t take them. But I still, I’m probably getting now four hours, if I’m lucky, five hours of sleep at the very most. That’s very rare. Usually it’s generally four hours.” (AML patient #110, male)
	“And, you know, I can go back to what my new normal life will be. And then the anxiety of that. What is my new normal life going to be? Is it going to be like my old life? Do I get it back or is it going to be completely different? And that part makes it difficult.” (AML patient #102, female)
	“So, their whole life is disrupted too. So, a lot of these patients are [thinking] about a lot of different things. Some patients think about their finances. Obviously, you know, ideally you don’t want to think about any of those stressful stuff. But, you know, their life is…you can’t just take them away. Some are thinking what’s going on in their homes and some are thinking on when this is going to be done, when they’re going to be able to go home and get done with this process. A lot of thoughts that goes through their mind and nobody is working.” (oncologist #304, male)
	“But some patients start thinking what’s going to be next for them. And that’s where most of the types I discovered they have depression to some degree. I always screen them for that. And I always tell them that if you tell me that you are happy, I would be surprised…And I would say there’s some degree of any depression and that would be depression, or you call it post-traumatic stress disorder, adjustment disorder, whether this is substantially contributing to their sleep…” (oncologist #305, female)

#### Theme 1: Continuation of poor sleep habits acquired during hospitalization

Adjustment difficulties were particularly noted by clinicians and patients during the isolation period. Clinicians identified that sleep and circadian rhythm disruptions stemmed predominantly from the hospital schedule, with the treatment administered and the vitals collected during hospitalization having lingering adverse effects on patients’ sleep. Upon discharge, patients continued to nap throughout the day, as they had in the hospital, to cope with their disrupted sleep, leaving them with a disrupted sleep/week rhythm. Clinicians also noted that patients had difficulty adjusting to no longer relying on sleep aids they had routinely used during their hospital stays, as these are less often prescribed during the isolation period.

#### Theme 2: Lingering treatment side effects

In addition to the lingering poor sleep habits, during this post-hospital isolation period, some patients continued to experience side effects from medications, cancer treatments, or both, with some patients developing graft-versus-host disease (GVHD). Patients and caregivers expressed that treatment side effects, such as GI symptoms, frequent urination, and pain, frequently woke patients up throughout the night. Clinicians also noted that steroids like prednisone cause insomnia and negatively affect patients’ abilities to fall asleep.

#### Theme 3: Challenges being in an unfamiliar, temporary place

During the isolation period, patients who live far from the transplant center typically stay at specialized transplant-designated housing so that they can remain close to the transplant center and the treatment team. Those patients often reported that environmental factors contributed to sleep disturbance. Patients recalled that being in an unfamiliar space that they could not control (e.g., choose/move furniture, decorate space) caused discomfort and disrupted sleep. This unfamiliarity interfered with routines and habits, disrupting behaviors that aid in building a sense of normalcy that patients sought.

#### Theme 4: Worry about adjusting to life post-transplant and the “new normal”

Having experienced a life-altering diagnosis and treatment regimen, patients, clinicians, and caregivers believed that anxiety in the form of “racing thoughts” and panic attacks interfered with patients’ sleep during the isolation period. These different presentations of anxiety (e.g., racing thoughts or panic attacks) were thought to be the result of adjustment difficulties, patient worries about routine expenses, home responsibilities, and health. Participants described in detail their life post-transplant and what would be their “new normal.” Clinicians echoed that many patients experienced this uncertainty about post-transplant life. They likened this anxiety to developing an adjustment disorder or other type of mental health concern, focusing too much on the future and the “new normal.”

### Home after isolation (post day 90)

While most patients’ sleep improved after returning home, all patients still reported some continued sleep disturbance. Three recurring themes described this period at home: (1) improved but ongoing sleep disturbance in the home environment; (2) lingering treatment toxicities; (3) continued worry about adjusting to life post-transplant and the “new normal.” Table 6 at the end of this section provides representative participant quotes aligned with the overarching themes observed post day 90.

**Table 6:** Main themes and representative quotes: home after isolation

Main themes	Representative quotes (participant, sex)
Theme 1: Improved, but lingering, sleep disturbance in the home environment	“I mean, certainly you sleep better in your own bed, in your own environment. And I was able to sleep much better, I think. Plus, you know, I was able to sleep with my wife. We were sleeping in separate rooms while we were here. And then we went back home and slept in the same bed, which was nice.” (CMML patient #105, male)
	“I mean, I went to bed last night at about nine thirty…but I didn’t get to sleep until after eleven thirty. So that’s my norm. I’m usually lying in bed a good hour to two hours before I finally fall asleep. And I was up by three thirty this morning because of the anxiety of coming here. Of course, I wake up, I’m not going back to sleep. And I did wake up. I wake up probably every hour or two and I don’t fall back asleep like a normal person would, you know? For some reason, my husband’s snoring, and it wakes me up. I can, you know, elbow him and go right back to sleep. That doesn’t happen anymore. It’s I’m awake.” (AML patient #102, female)
	“He seems to hear everything. He’s a lighter sleeper, even though he’s got the Ambien now. He’s a lighter sleeper than he ever was before. He complains I’m being loud all the time. So yeah, if I’m being loud…Yeah, he just doesn’t sleep as sound as he once did.” (AA caregiver #206, female)
Theme 2: Lingering treatment toxicities	“Well, the thing that really causes me to not sleep that well or to fall asleep is because of the way I feel. I could feel the nausea. I could feel like my stomach cramps. I feel like I just don’t feel sleep with me this far.” (ALL patient #104, male)
	“He’s sleeping a little bit better, but he still wakes up in the middle of the night to use the bathroom. He wakes up most of the time. Sometimes, when he gets up in the morning, [he says] ‘Oh, I don’t feel good, I feel sick.’ Stuff like that, but he is not as bad as when he had the transplant.” (ALL caregiver #201, female)
	“But others are legit post-cancer and post-therapy issues, pain issues. A lot of our women are forced into menopause with the therapy and the transplants. So, they’re having a lot of hormonal issues, which comes with the shortened sleep cycle.” (interventional radiology technician #303, female)
Theme 3: Continued worry about adjusting to life post-transplant and the “new normal”	“But then when we’re back home and we must face life going forward, there’s a lot of stress involved with that. I’m back at work now. I’ve been working part time and so on. And I feel like I can’t do as well as I did before. And that makes it really hard.” (ALL patient #107, female)
	“I’m in my own bed and home around the family and such and my four-legged children. So that part was nice, but I think that’s when some of the anxiety got worse, because now I’m home. And now what?” (AML patient #102, female)
	“… it took me 18 months, almost two years post-transplant right now. And I just stopped thinking about relapse. It’s almost still a point of obsession in the very beginning. It was real, real bad…Any little thing. I’d be worried that cancer is coming back. Cancer is coming back. It drove me nuts.” (AML patient #110)
	“They’re also reporting some degree of anxiety that is preventing them from sleeping. So, not necessarily there’s some medical reason why they can’t go to sleep. But usually there is an anxiety or depressive component, predominantly anxiety, that’s keeping them awake at all hours of the night for which they need something to kind of shut the brain off because they are not able to do it.” (oncologist #306, female)

#### Theme 1: Improved, but lingering, sleep disturbance in the home environment

Access to their own beds and home environment improved most patients’ sleep upon returning home. The physical and psychosocial supports at home, such as family, sleeping with their spouses, and having a private space, helped patients feel more comfortable. However, some patients also reported that, despite returning home, they still struggled with sleep and noticed changes in their typical sleep patterns. Caregivers also commented on this change in their loved one’s sleep behavior post-HCT noticing a change in patient sensitivity to their environments.

#### Theme 2: Lingering treatment toxicities

In addition to having sleep disturbances from the home environment, many of the patients continue experiencing lingering toxicities from the medication and cancer treatment. For example, GVHD symptoms or frequent nocturia continued after returning home. Medications also appeared as a common stressor for caregivers, with many caregivers believing lingering medication toxicities, such as GI issues, interfered with patients’ sleep. Many of the clinicians also mentioned chronic GVHD, early onset menopause for women, and lasting side effects as disruptive health issues following the patients’ time at home. One clinician explained that, at day 90, they begin to see about half the patients return to their normal sleep schedules and the other half continues to experience sleep disturbance.

#### Theme 3: Continued worry about adjusting to life post-transplant and the “new normal”

Finally, patients continued to experience adjustment difficulties after day 90. They explained their self-realization that they now needed to face life moving forward, but were unsure what it entailed, leading to continued worry. Most patients discussed anxiety and stress regarding health, financial toxicity, and returning to work as significant barriers to quality sleep. They reported trying to return to their “new normal” as they tried rebuilding daily routines. Fear of a potential relapse was also a prominent concern, as patients expressed that racing and intrusive thoughts around relapse either kept them awake or kept them from returning to sleep after waking during the night.

While most caregivers did not mention how anxiety interfered with patients’ sleep, the clinicians were very aware that patients struggled with anxiety, with most reporting anxiety as a barrier to sleep. A few clinicians even explained how patients can develop PTSD from the transplant process, which can inhibit patients’ ability to either fall asleep or return to sleep after awakening during the night.

## Discussion

This study qualitatively explored patterns of sleep disturbance among allogeneic HCT recipients throughout the treatment journey, from the perspectives of patients, caregivers, and clinicians. Changes in patients’ sleep patterns were persistent and multifactorial, noted at clinically relevant time periods: during hospitalization, during the isolation period, and after returning home. Patients, clinicians, and caregivers all noted that hospitalization was the time period with the most sleep disturbance, serving as a primary catalyst for dysregulating the sleep-wake cycles, which continued through recovery. The time spent in the hospital provides a potential target for future sleep interventions and advocating for “sleep-friendly” hospitals, aiming to minimize disruptions during hospitalization [25, 26]. Another key point of intervention is once patients return home, focusing on interventions that enhance sleep hygiene and restore disrupted sleep-wake cycles, helping patients adjust to life post-transplant.

Consistent with the current literature on sleep disturbance among HCT recipients, our participants had continued sleep disturbance after transplant [11, 12, 27]. Our findings indicate that the reasons for the sleep disturbances observed during isolation were extensions of those experienced during hospitalization. Patients continued with maladaptive sleep habits, such as daytime napping, acquired due to their disrupted schedule, and continued having difficulty falling and staying asleep due to their anxiety about their future or their “new normal.” Clinicians also highlighted sleep aids as an additional reason for continued sleep disturbance, noting that patients became reliant on them during their hospital stays to cope with their disrupted sleep schedule. Furthermore, the unfamiliarity of the specialized transplant housing, coupled with treatment side effects, contributed to the lingering sleep disturbance.

Upon returning home, patients experienced some improvement in their sleep but also noticed lingering struggles with sleep quality and difficulty falling and staying asleep. Echoing our findings, a study by Rentscher et al. [12] found that 43.2% of HCT recipients in their study had sleep disruption as defined by the Pittsburgh Sleep Quality Index (> 5) at day 100 post-HCT. The caregivers shared this concern regarding lingering sleep disturbances at home due to prolonged treatment toxicities, and patients’ fears of having maladjustment to life at home.

Currently, the majority of studies rely on self-report scales to provide quantitative measures to describe the sleep experience of HCT recipients [12, 28]. This study contributes to the literature by mapping the sleep trajectory for HCT recipients, incorporating multiple perspectives (patients, caregivers, and clinicians) to help unpack the complexity of sleep issues providing context for the quantitative findings observed in the literature. These qualitative findings also help identify potential intervention points among HCT recipients before chronic sleep issues become entrenched. As previously mentioned, two potential intervention points include the following: (1) minimizing disruptions during hospitalization, or (2) targeting once patients return home to enhance sleep hygiene and restore a disrupted sleep-wake rhythm.

### Strengths and limitations

This study provided a deeper understanding of the multiple contributing factors to sleep disturbance among a community significantly impacted by poor biobehavioral symptoms, HCT recipients. While this study begins filling a gap in the literature and advances understanding of the course and influences of sleep disruption in HCT recipients from multiple perspectives, there are some limitations to note. First, while this study offers unique perspectives of patients, caregivers, and clinicians, its small sample size may limit the generalizability of our findings to other cancer centers or all HCT recipients. The patients and caregivers were also post-hospitalization, potentially introducing recall bias. There was also limited racial/ethnic diversity in our sample, which may limit its applicability across different racial/ethnic groups. Despite the limited sample size and the interview timing within the transplant trajectory, we identified recurring themes regarding HCT recipients’ persistent sleep disturbances that could inform future work. Furthermore, although the interviews were conducted in 2018, the findings remain relevant and applicable to the current literature and future research.

## Conclusion

This qualitative study helped expand research on biobehavioral symptoms among allogeneic HCT recipients and unpack the multifaceted contributors to lingering sleep disturbances from multiple perspectives. One avenue of future work should focus on interventions that promote “sleep-friendly” hospital procedures to limit sleep disruptions for HCT recipients [25, 26]. Additionally, future work should focus on creating interventions with low participant burden, such as mHealth interventions, to help reduce sleep disturbances among HCT recipients and support their QOL upon returning home. Some studies are already being conducted to examine the effectiveness of app-based interventions for insomnia and sleep disturbance for the general population and those impacted by cancer [29, 30]. It is crucial to utilize the patient, clinician, and caregiver perspectives presented in this work to advocate for improved hospital procedures and targeted interventions to improve HCT patients’ sleep and overall QOL.

## Data Availability

Data analyzed during the current study are available upon reasonable request.
